# Analgesic efficacy of ultrasound-guided subcostal transversus abdominis plane block

**DOI:** 10.1097/MD.0000000000006309

**Published:** 2017-03-10

**Authors:** Jianfeng Ma, Yifei Jiang, Shiyi Tang, Benfu Wang, Qingquan Lian, Zuokai Xie, Jun Li

**Affiliations:** a Department of Anesthesiology; b Medical Records Room, The Second Affiliated Hospital and Yuying Children's Hospital of Wenzhou Medical University, Wenzhou, Zhejiang, China.

**Keywords:** analgesia, levobupivacaine, regional anesthesia, subcostal TAP, zone

## Abstract

**Background:**

To evaluate the analgesic efficacy on defined areas of the abdomen and back after ultrasound-guided subcostal transversus abdominis plane (TAP) block using 0.25% levobupivacaine 0.5 mL/kg.

**Methods:**

Twenty patients undergoing elective laparoscopic cholecystectomy, between 20 and 60 years of age with operative time <1 hour, received subcostal TAP block using 0.25% levobupivacaine 0.5 mL/kg on the left side. Surgery started after 1 hour of observation. Sensory assessment was undertaken using pinprick and 75% ethyl alcohol at 10, 20, 30 minutes, 1, 3, and 12 hours after TAP block at 19 testing zones that were divided by anatomic landmark lines on the abdomen and the back. Efficacy of zone was defined as loss of cold temperature sensation or loss of pinprick pain sensation in more than 50% patients in that testing zone. Duration was determined by analgesia and loss of temperature sensation beginning within 30 minutes of TAP block placement lasting until time points of 1, 3, and 12 hours. All of the testing zones were divided as Group I effective at 20 minutes in less than 50% patients (0%–50%), Group II 50% to 70% patients, Group III 70% to 90% patients, and Group IV 90% to 100% patients.

**Results:**

Twenty patients meeting the study requirements were included. At each time point, the efficacies among 4 groups were significantly different. Subcostal TAP had good efficacy and stable duration in zones 1, 2, 3, 5, and 6.

**Conclusion:**

Subcostal TAP block with 0.25% levobupivacaine 0.5 mL/kg dose provided effective analgesia in the anterior abdominal wall between medioventral line to anterior axillary line except the lateral upper abdominal region.

## 1 Introduction

Transversus abdominis plane (TAP) block is a new technique of regional anesthesia, which reduces the pain derived from abdominal wall incisions,^[1–3]^ decreases general anesthesia requirements,^[4]^ and increases hemodynamic stability.^[5,6]^ Previous reports have demonstrated that distribution and diffusion of local anesthetics in the plane of the transversus abdominis muscles vary by the location of block placement^[7]^; compared to the midaxillary line approach, the subcostal approach TAP block can provide more reliable analgesia of the abdominal area above the umbilicus.^[8,9]^ But the time required for anterior and posterior distribution of local anesthetic block of the abdominal dermal regions is not yet clear. This study was designed to investigate the analgesia efficacy, by defined dermal zones, of ultrasound-guided subcostal TAP block using 0.25% levobupivacaine 0.5 mL/kg.

## 2 Methods

After obtaining approval from the Ethics Committee at The Second Affiliated Hospital of Wenzhou Medical University (IRB 2014-02) and written informed patient consent and registered in Chinese Clinical Trial Registry (ChiCTR-OOC-14005350 10/19/2014), a convenience sample of 20 patients was recruited for this study. Inclusion criteria were American Society of Anesthesiologists grade I or age 18 to 60 years and elective laparoscopic cholecystectomy with anticipated operation time less than 1 hour. Exclusion criteria were neuropsychiatric disease, substance addiction, and recent use of sedative or analgesic drugs. All TAP blocks were placed by a physician with 3 years’ nerve block experience. An observer who did not know the study, group assignment was responsible for assessment of the analgesic efficacy.

Electrocardiogram, noninvasive blood pressure, and oxygen saturation were routinely monitored before TAP block. A 12-MHz high-frequency linear array probe (Sonosite, Bothell, WA) was positioned on the anterior abdominal wall immediately inferior to the costal margin. A 22-ga needle (Braun, Aschaffenburg, Germany) then was advanced to 2 to 3 cm lateral to the aponeurosis of the external oblique muscles, internal oblique muscles, and transversus abdominis in the TAP. Saline 0.5 mL was injected to determine the position of the transverse fascia, followed by 0.25% levobupivacaine 0.5 mL/kg TAP block in the left side. One hour after the completion of the TAP block, general anesthesia was induced with propofol 2.5 mg/kg, sufentanil 0.25 μg/kg, and rocuronium 0.8 mg/kg followed by endotracheal intubation. Anesthesia was maintained with propofol and remifentanil. Local anesthesia with 0.375% ropivacaine was injected by the surgeon at each laparoscopic puncture site (umbilicus 2 mL and subxiphoid 0.5 mL) and in the incision at right costal margin (0.5 mL) after surgery.

Sensory assessment was undertaken using 75% ethyl alcohol swab and pinprick at 10, 20, 30 minutes, 1, 3, and 12 hours after TAP block completion at specific dermal zones that were divided by anatomic landmark lines on the abdomen, the back, and the thigh. Effective analgesic efficacy was defined as loss of cold temperature sensation to alcohol swab or loss of pinprick pain sensation compared with the nonblocked area.

**Figure 1:**
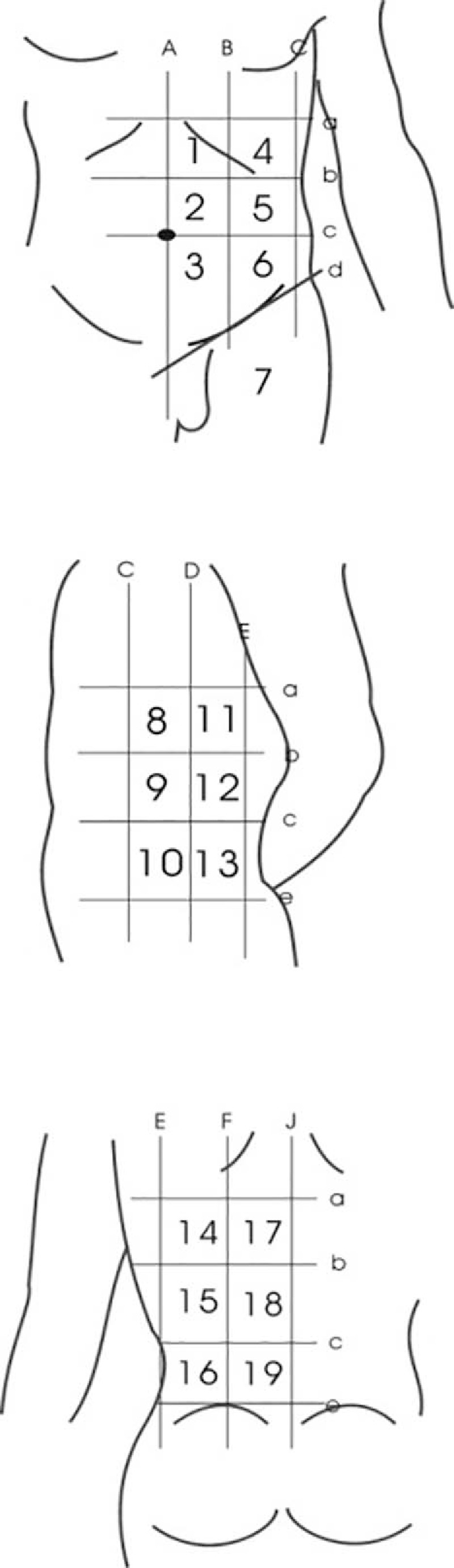
Eighteen dermal zones described by horizontal lines at xiphoid (a), costal margin (b), umbilicus (c), anterior superior iliac spine to the pubic symphysis line on the abdomen (d), and the back (e); and vertical lines at midline (A), mid-clavicular line (B), anterior axillary line (C), mid-axillary line (D), posterior axillary line (E), infrascapular line (F), and back midline (J). Zone 7 is upper 1/3 part of the front thigh.

The 19 specific dermal zones used in this study (Fig. 1) were described by vertical lines at the midline (A), mid-clavicular line (B), anterior axillary line (C), mid-axillary line (D), posterior axillary line (E), infrascapular line (F), and back midline (J); 4 horizontal lines at the xiphoid level (a), 12th costal level (b), the umbilical level (c), the anterior superior iliac spine to the pubic symphysis line (d), and extension to the back to the anterior superior iliac spine level (e). An additional zone was defined as the upper 1/3 of the front thigh (Fig. 1).^[10]^

Four sensation measurement points evenly distributed in each zone were used as the sensory assessment tool, when the incision is located in the zone, the sensation points should be more than 1 cm away from the incision. A dermal zone was considered effectively blocked by the TAP block, if there were 2 or more sensory assessment points in the zone that showed loss of temperature sensation to alcohol swab or pain sensation to pinprick. The zone was considered as negative (not effectively blocked) if less than 2 points showed pain or temperature sensory reduction.

The time from TAP block completion to block level (at least 2 vertically consecutive zones, e.g., zones 1 and 2, or zone 4 or 5) appeared was recorded. Failure to block was determined if there were less than 2 vertically consecutive zones block, if so subjects were excluded.

### 2.1 Data preparation and statistical analysis

In this study, the median effective dose for each zone was defined as effective analgesia obtained in 50% of patients (i.e., the same zone appeared positive in 10 of 20 patients); when more than 50% patients had effective analgesia at the same zone, it was defined effective. Based on the effective rate at 20 minutes time point, 19 zones are divided into 4 groups. Group I was defined as 0% to 50% effective (effective in less than 50% patients), Group II as 50% to 70% effective, Group III as 70% to 90% effective, and Group IV as 90% to 100% effective. Stable duration of each individual zone was determined by more than 50% effective rate of at least 2 time points of 10, 20, and 30 minutes and at least 2 time points of 1, 3, and 12 hours.

Statistical analysis was performed using SPSS 22.0 (SPSS Inc, Chicago, IL) statistical software, measurement data are expressed as mean ± standard deviation. Hemodynamic data were compared using repeated measures analysis of variance. The effective rates among the groups were compared using chi-square test with *P* < 0.05 considered statistically significant. The effective rates between the 2 groups were compared using chi-square test and Fisher exact test (adjusted for multiple comparisons), *P* < 0.0083 was considered statistically significant.

## 3 Results

Twenty-four patients aged between 24 and 57 years of either sex, BMI 18.7 to 24.6, met the inclusion criteria and were enrolled in the study. One patient was excluded due to failed TAP block, and 3 patients were withdrawn due to operative time exceeding 1 hour. Thus, 20 cases were analyzed.

**Figure 2:**
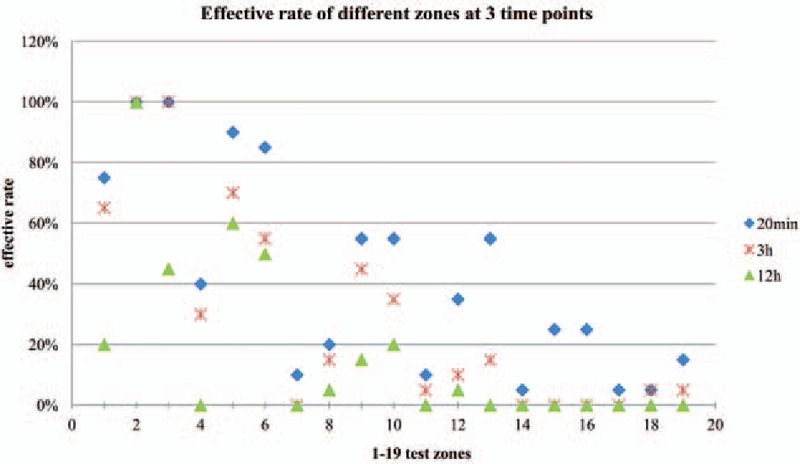
Result of effective rates of 19 dermal zones on the left abdomen, the back, and the thigh in 20 patients at 3 time points. Effective rates of zones 2, 3, 5, and 6 were always higher than 50%. Among zones 1, 9, 10, and 13, the effective rates were higher than 50%, but not all of the 3 time points, all the other zones never had effective rates higher than 50%.

**Figure 3:**
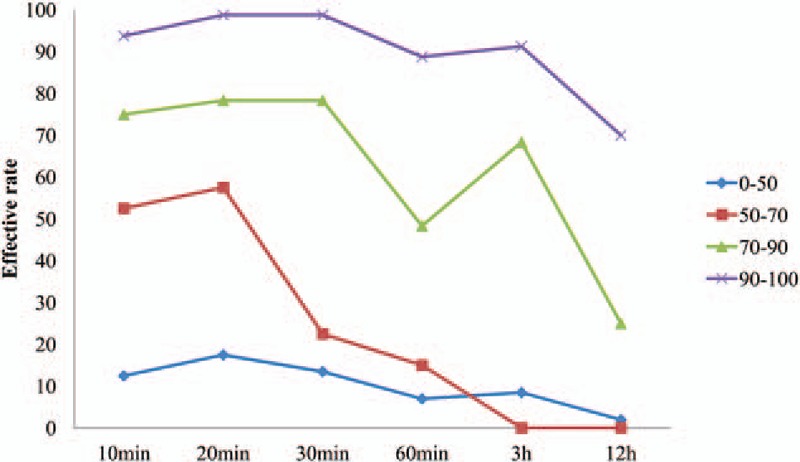
Results of the effective rates of 4 groups at 6 time points are shown. Effective rates of Group IV were always higher than 50%; effective rates of Group III were higher than 50% except 12 hours time point; in Group II, the effective rates were higher than 50% at 10 and 20 minutes, but lower than 50% at other time points; Group I never had effective rate higher than 50% at any time.

The average onset time of their block level appeared was 1.01 ± 0.02 minutes. The zones were divided into 4 groups by effective rates at 20-minute time points, the effective rates are listed in Table 1. The effective rates of different zones at 20 minutes, 3, and 12 hours are shown in Fig. 2; effective rates of zones 2, 3, 5, and 6 were always higher than 50%, especially zone 2, for which effective rate was 100% at 20 minutes, 3, and 12 hours after TAP. Among zones 1, 9, 10, and 13, the effective rates were higher than 50%, but not all of the 3 time points, all the other zones never had effective rates higher than 50%. The effective rates of different groups at 6 time points are shown in Fig. 3; effective rates of Group IV were always higher than 50%, even at 12 hours, it is 70%; effective rates of Group III were higher than 50% except 12-hour time point; in Group II, the effective rates were higher than 50% at 10 and 20 minutes, but lower than 50% at other time points; Group I never had effective rate higher than 50% at any time point. Subcostal TAP had good efficacy and stable duration in zones 1, 2, 3, 5, and 6.

**Table 1:**
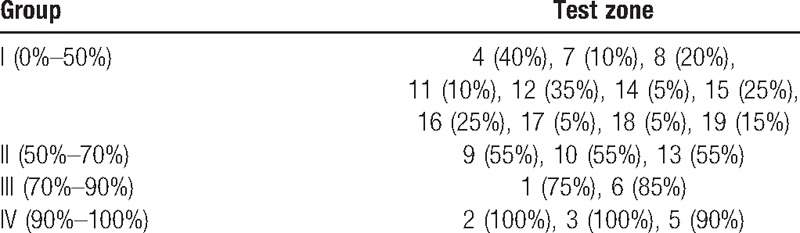
Effective rates of each group 20 minutes after TAP.

The effective rates at the 6 time points among the 4 groups were significantly different (*P* < 0.05) (Table 2), also significantly different (*P* < 0.0083) when compared in pairs, except Groups II and III at 10 or 20 minutes, Groups III and IV at 20 or 30 minutes. Hemodynamics all time points had no significant difference among the groups (Table 3).

**Table 2:**
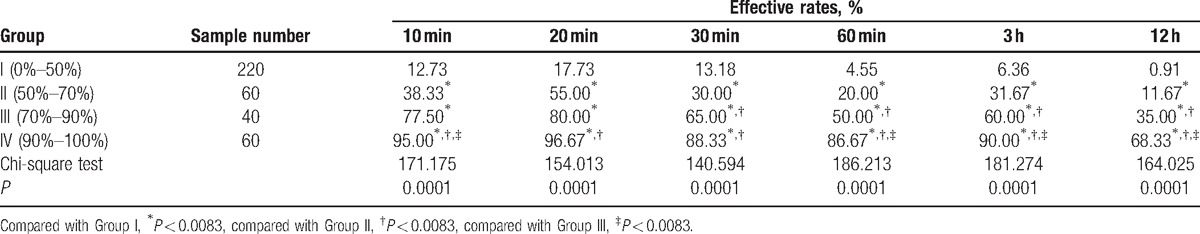
The effective rates at different time points by groups.

**Table 3:**
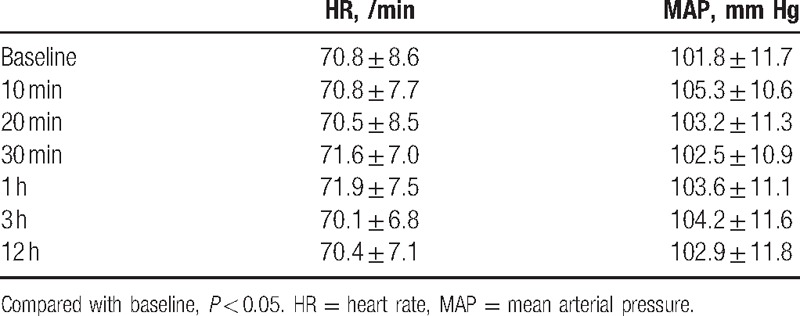
Hemodynamics at different time points.

## 4 Discussion

This is the first study that has divided the abdominal region and the back into small dermal zones to assess the analgesic efficacy of the subcostal TAP block. Using this innovative method, we are able to accurately assess the blockade efficacy on different areas of the abdominal region and the back and also predict postoperative analgesic efficacy. Effective TAP was defined as either loss of cold temperature sensation or loss of pain sensation. The median effective dose for each zone, defined as effective analgesia, was obtained in 50% of patients. This method is very sensitive in detecting the analgesic efficacy of dermal zones and shows the precise block area of the abdominal region and the back. The time to the peak plasma concentration of levobupivacaine is 30 minutes after injection.^[11]^ The 6 assessment time points are divided into 3 time points before 30 minutes after injection, and 3 time points after 30 minutes. We were looking for an area located on the abdominal region and the back that results in effective analgesia both before and after the anesthetics reach peak plasma concentration so that we may utilize TAP method in the postoperative analgesia of abdominal/back surgery more effectively.

Our results showed that the subcostal TAP block had the longest duration of analgesia at zones 1, 2, 3, 5, and 6, indicating that the subcostal TAP block was effective and long-lasting at the anterior abdominal wall, from the midline to the anterior axillary line but not including the lateral epigastric area. Studies have shown that subcostal TAP can decrease postoperative narcotics use,^[2,12,13]^ which plays an important role in multimodal analgesia treatment. The incision area is the upper anterior abdominal wall in these studies, which is consistent with our results that showed that the best area of effective analgesia is at the anterior upper abdominal wall. For zone 4, located at the lateral epigastric area, the effective rates at 20 minutes, 3, and 12 hours are 40%, 30%, and 0%—all lower than 50%, indicating that subcostal TAP block has some analgesic efficacy but not enough for surgery and postoperative analgesia. This result partly contrasts with work by Milan et al^[14]^ and Maeda et al,^[15]^ who found that subcostal TAP had sufficient analgesic efficacy during liver transplant surgery. This could be relevant to the needle tip at the transverse fascia located closer to midline. Subcostal TAP assisted by small-dose fentanyl also has been successfully performed in open gastrostomy in 5 patients.^[16]^ Only 1 patient needed local anesthetic injection to the superior end of the incision. The incision area was at the upper midline, which falls within zones 1 and 2 by our definition. Our results showed that the effective rates of zone 2 at all the time points were 100% and zone 1 at 20 minutes and 3 hours points were 75% and 65%, as the same as this article.

It has been shown that the block at levels T6 to T7 can be obtained by the subcostal approach; rarely, the block can reach L1 level.^[17,18]^ This is consistent with the results of our study. Zones 1 and 4 are located at levels T6 to T8, zone 7 is located at level L1. Though zone 7 showed analgesia in some patients, the effective rate was only 10% at 20 minutes and 0 at 3 and 12 hours. The block can reach L1 level sometimes but it is very rare and has no clinical significance. There is no previous study showing the analgesic efficacy of subcostal TAP block at the lateral abdomen or the back. In our study, the corresponding areas of the lateral abdomen were zones 8, 9, 10, 11, 12, and 13. They all had effective analgesia at different time points. The effective rates of zones 9, 10, and 13 at 20 minutes were higher than 50%, indicating that subcostal TAP had analgesic efficacy on the lateral abdomen. Zones 14, 15, 16, 17, 18, and 19 were located at the back; analgesia lasted till 3 hours points in 1 patient. Though the effective rates were very low, it means in some patients that the block can reach to the back. This could be caused by the large amount of drugs infiltrated and diffused toward the back and then blocked the peripheral nerve; the drug may also diffuse into the paraspinal area and produce unilateral a block at the abdomen and the back. This is consistent with the corpse staining that was reported by Carney et al.^[19]^

Most of the previous studies^[7,17,18]^ only reported and analyzed the comparison of different plane levels, but never analyzed the diffusion of the block from midline to the back. If we use TAP block for postoperative analgesia purpose without knowing the diffusion pattern, for example, using TAP block on an upper lateral abdominal incision, it likely to cause poor analgesia results. Unlike epidural anesthesia, which will have a block effect depending on the level it is applied, the exact mechanism of TAP block is still unclear. The mechanisms can be several. Large surface spread through subcostal approach TAP caused more nerves blocked^[20]^; the anterior abdominal wall is innervated by the multiple conjoined segmental thoracolumbar nerves, which branch and communicate widely at multiple locations as intercostal plexus, TAP plexus, rectus sheath plexus,^[21]^ or blockade at paraspinal area.^[19]^ Our results showed that there are big differences at different regions of the abdomen and the back, and it also shows good blockade duration at the area that showed good analgesic efficacy in a short time after TAP block. The areas with best efficacy are mainly located at the anterior abdominal wall, the lateral abdominal wall is less effective to TAP block, and almost no analgesic efficacy at the posterior abdominal wall, indicating that subcostal TAP block diffused as patchy pattern and gradually weakened from anterior to the posterior of abdominal wall.

Midline incision and lateral rectus incision are more common clinically, but some operations such as the abdominal flap or skin graft are related to the lateral abdomen. Our study reveals how the subcostal approach diffuses laterally, which can be a guide to provide anesthesia and pain management more precisely. In addition, the results show hemodynamic stability after the subcostal approach TAP block with 0.25% levobupivacaine 0.5 mL/kg, it is a good choice for anesthesia and analgesia plan in elderly patients and cardiovascular instable patients, which is consistent with previous studies.^[5,6,22]^

However, due to the small sample size in our study, further studies are still needed to confirm the reliability. Moreover, in order to avoid the pain at surgical incision interfering with the test measurements, we only measured the area at left abdomen and left back, meanwhile local anesthesia was injected by the surgeon at laparoscopic puncture site and the incision at right costal margin after surgery. Although we measured the sensory at more than 1 cm away from the incision, the local anesthesia application could still effect the assessment of the zone in which it is located.

In summary, subcostal approach TAP block with 0.25% levobupivacaine 0.5 mL/kg has effective analgesia of long duration at the anterior abdominal wall from the midline to axillary line except the lateral upper abdominal region.
